# Relationship Between Schmidt Hammer Rebound Hardness Test and Concrete Strength Tests for Limestone Aggregate Concrete Based on Experimental and Statistical Study

**DOI:** 10.3390/ma18061388

**Published:** 2025-03-20

**Authors:** Esra Tugrul Tunc

**Affiliations:** Civil Engineering Department, Engineering Faculty, Firat University, 23119 Elazig, Turkey; esratugrul@firat.edu.tr

**Keywords:** concrete strength, Schmidt hammer rebound hardness, destructive testing, non-destructive testing, statistical methods

## Abstract

This study investigated the mechanical properties of concrete specimens produced with a limestone aggregate through laboratory testing. Destructive tests, specifically concrete compressive strength and splitting tensile strength tests, were conducted. Additionally, the Schmidt hammer rebound hardness test, a non-destructive method, was performed on the same specimens. The experimental results, obtained from varying water-to-cement and limestone aggregate-to-cement ratios, yielded the following ranges: compressive strength from 23.6 to 42.6 MPa, splitting tensile strength from 3.2 to 5.1 MPa, and Schmidt hammer rebound values from 18 to 43 N. The correlation between the non-destructive and destructive test results was analyzed experimentally and statistically. Utilizing the experimental data, statistical models were developed, resulting in equations with a high determination coefficient (R^2^ > 0.95) for accurately predicting concrete compressive and splitting tensile strengths. This approach offers the potential for significant labor and time savings in the production of sustainable conventional concrete that meets relevant standards. Furthermore, it aims to facilitate the estimation of concrete strength in existing structures.

## 1. Introduction

The recent devastating earthquakes in Turkey, the location of this study, have highlighted the critical issue of poor concrete quality and durability in collapsed buildings. Consequently, it is imperative that the concrete used in new constructions, particularly in regions situated along active fault lines, adheres to stringent quality standards [1,2]. Therefore, the assessment of concrete strength, especially in older structures, through both destructive and non-destructive testing methods is of paramount importance [3,4]. The practicality of non-destructive tests, characterized by their ease of application, rapid on-site results, and the absence of structural damage, significantly enhances their value in concrete evaluation [5,6].

Non-destructive testing methods have become increasingly vital for concrete quality control and structural condition assessment, as they offer the advantage of evaluating concrete integrity without causing damage [7,8]. Destructive testing of concrete structures can lead to deterioration and structural compromise. Furthermore, these methods present inherent challenges in application and accurate interpretation. Techniques such as coring or vertical drilling, which inflict structural damage, are not only complex and risky but often yield specimens unsuitable for reliable laboratory analysis due to significant damage [9,10]. Consequently, enhancing the accuracy and reliability of non-destructive testing methods is crucial, facilitating their broader adoption as a preferred alternative to destructive testing.

The successful implementation of non-destructive testing methods in structural engineering hinges on a comprehensive understanding of material properties and the fundamental principles governing their application [11,12]. Enhancing defect detection capabilities necessitates that application engineers investigate the interaction of diverse concrete constituents with various non-destructive testing techniques. Each non-destructive testing method, including ultrasonic testing, radiographic testing, magnetic particle inspection, and eddy current testing, possesses distinct limitations and requirements [13,14]. Consequently, ensuring the selection of the most suitable non-destructive testing method for a given application requires precise characterization of material properties and meticulous equipment calibration [15,16].

A significant challenge in non-destructive testing methods lies in the inherent variability of data measurements [17,18]. When evaluating the material condition and alterations in material properties, the primary focus is typically on variability induced by damage, designated as the “signal”. Conversely, all other sources of variability, irrespective of origin, are classified as “noise”. This noise can stem from the material’s intrinsic variability or the measurement process itself [19]. The reliability of non-destructive testing methods is susceptible to numerous parameters, including source–receiver distance, measurement grid spacing, specimen geometry, boundary conditions, and the size, location, and nature of discontinuities [20,21]. Furthermore, alterations in concrete microstructure, such as those caused by environmental fluctuations, variations in concrete constituent properties, and concrete porosity, can introduce measurement errors. Consequently, it becomes challenging to directly correlate observed changes in measured properties, such as wave velocity or electrical resistivity, to a specific physical cause [22].

Concrete compressive strength, defined as the maximum stress concrete can withstand under compressive loading, is a fundamental mechanical property used for concrete classification [23,24,25,26]. While compressive strength can be assessed at various curing durations, the standard measurement is typically conducted after 28 days of curing [27,28]. This property serves as a strong indicator of other concrete characteristics, rendering it a critical parameter in structural evaluations [29,30]. Factors significantly influencing concrete compressive strength include the material properties, water–cement ratio, aggregate–cement ratio, and curing methodology [31].

Non-destructive testing methods are crucial tools for assessing structural deficiencies in concrete specimens and structures, both during and after the casting process, without compromising their integrity [32,33]. These methods represent a dynamic and evolving field of study. Due to concrete’s inherent heterogeneity, the development of reliable non-destructive testing techniques requires extensive research and refinement. Nevertheless, many of these methods have been standardized by organizations such as ASTM, ISO, and BSI [34,35]. Among the most widely employed non-destructive testing methods for indirectly evaluating concrete strength are the Schmidt hammer rebound hardness test, the Windsor Probe Penetration Test Method, Radiographic Testing, Ground Penetrating Radar, Ultrasound Pulse Velocity Testing, and Ultrasonic Tomography.

In 1948, E. Schmidt introduced the Schmidt hammer as a non-destructive tool for estimating concrete compressive strength [36]. Today, the Schmidt hammer rebound test, gaining increasing prevalence, offers a non-destructive alternative to destructive methods like core sampling for assessing concrete compressive strength. However, the accuracy of this method is contingent upon rigorous calibration [37,38].

For accurate Schmidt hammer measurements, it is essential that the concrete surface being tested, as well as the surface used for correlation, is clean, flat, and dry. Deviations from these conditions can introduce errors ranging from 60 to 70% [39,40]. Individual application of non-destructive testing methods is generally considered insufficient for precisely determining the concrete compressive strength of existing structures. However, their reliability can be enhanced through correlation techniques. Specifically, it has been demonstrated that the actual concrete compressive strength can be estimated from Schmidt hammer rebound readings by applying appropriate correlation coefficients [41].

In contemporary civil engineering, the efficient and accurate on-site determination of concrete properties, including quality, strength, corrosion, damage, and defect detection, is paramount. Monitoring time-dependent changes in concrete properties, identifying detrimental impacts on strength and durability, detecting potential defects, and conducting comprehensive damage assessments are crucial for implementing timely preventative measures. The non-destructive nature of these testing methods allows for repeated evaluations without structural compromise, leading to significant time and cost savings. Furthermore, the portability and ease of application of non-destructive testing equipment minimize labor costs and enable immediate access to test results [42,43].

Owing to certain limitations associated with the uniaxial compressive strength test, the Schmidt hammer test has been employed for assessing rock hardness since 1966 [44]. Initial research conducted between 1966 and 1980 focused on various lithologies, including basalt, diabase, and dolomite, aiming to establish empirical relationships between Schmidt hammer rebound values and uniaxial compressive strength through experimental data. These studies consistently demonstrated the utility and preference of the Schmidt hammer in engineering practice, primarily due to its portability, compactness, and lightweight design [45,46]. Subsequently, research spanning from 1981 to 1993 further substantiated the advantages of the Schmidt hammer test, leading to its official recommendation by the International Society for Rock Mechanics (ISRM) [47] for rock material hardness assessment. Consequently, numerous empirical equations for estimating uniaxial compressive strength have been developed, gaining widespread acceptance and further development by numerous researchers [48,49]. Since 1994, with the advancement of computer science, the development of statistical and artificial intelligence-based computational techniques for predicting rock properties using Schmidt hammer data has continued to proliferate [50,51,52].

While the Schmidt hammer test has provided notable advancements in the assessment of uniaxial compressive strength, persistent challenges remain. Although empirical formulas are frequently employed for in situ evaluations due to their inherent simplicity, their validation has been limited by restricted experimental datasets and a narrow range of lithological variations. Consequently, there exists a critical need for these formulas to be substantiated by comprehensive experimental data. Furthermore, the principal novelty of the present study lies in its focus on the testing of concrete specimens fabricated within a controlled laboratory setting, a departure from prior research that predominantly investigated rock materials.

Extant literature reveals a substantial body of research concerning both destructive and non-destructive concrete strength assessment. However, a notable gap persists in the experimental and numerical investigation of the correlation between these testing methodologies, specifically for conventional concrete employing limestone aggregates. This study endeavors to address this deficiency by proposing an empirical formulation for predicting concrete strength, utilizing the Schmidt hammer rebound value (R-value) obtained during both the fresh concrete production phase and in existing structural elements. Consequently, this research aims to contribute to the advancement of the field by enabling the high-accuracy determination of destructive test outcomes, namely, compressive strength and splitting tensile strength, through non-destructive means.

The principal contributions of this study encompass the examination of conventional concrete incorporating limestone aggregates, the development of empirical formulations for predicting destructive strength values of concrete with defined mix proportions, and the establishment of a formulation predicated on the Schmidt hammer rebound value (R-value) of existing structural concrete. These aspects collectively represent a novel approach. The experimental methodology distinguishes itself from prior research by achieving standardized concrete strength using limestone aggregate without admixtures, systematically varying the water–cement and aggregate–cement ratios, and correlating the resultant strength with Schmidt hammer test outcomes. The experimental design and statistical analysis herein aim to demonstrate the efficacy of the Schmidt hammer test as a non-destructive and readily implementable strength assessment technique.

This study investigated the correlation between destructive and non-destructive testing methods by performing concrete compressive strength, splitting tensile strength, and Schmidt hammer rebound tests on a series of limestone aggregate concrete specimens with varying mix proportions. Statistical models were developed to establish relationships between the strength parameters obtained from destructive tests and the Schmidt hammer rebound values, considering different water–cement and aggregate–cement ratios. The primary objective was to evaluate the efficacy of the Schmidt hammer rebound test as a reliable, non-destructive method for predicting the strength of limestone aggregate concrete. Through the combined experimental and statistical analysis, this research aims to validate the practical applicability of the Schmidt hammer rebound test, thereby promoting its safe and efficient utilization in engineering practice.

## 2. Materials and Methods

### 2.1. Materials

**Figure 1:**
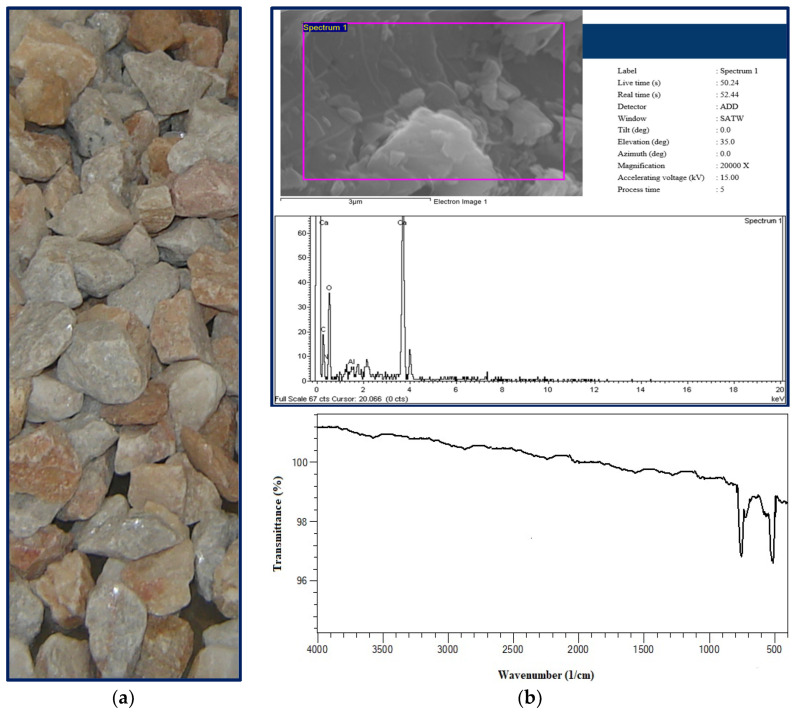
Tested limestone aggregate: (**a**) natural view, (**b**) SEM image/EDX analysis and FTIR analysis.

This experimental investigation utilized limestone aggregates with a maximum nominal size (*D_max_*) of 16 mm, sourced from the Elazığ province of Turkey (Figure 1a). The selection of 16 mm for *D_max_* was predicated on the sieve analysis results, which indicated that 100% of the aggregate passed through the 16 mm sieve, and to assess the influence of maximum aggregate particle size on concrete properties. The limestone aggregates were subjected to sieving, and the retained material on each sieve was segregated into five distinct size fractions: 0–1 mm, 1–2 mm, 2–4 mm, 4–8 mm, and 8–16 mm. To maintain consistent granulometry across all concrete mixtures, the aggregate proportions were determined according to Fuller’s parabola [53,54], resulting in the following composition: 25% for 0–1 mm, 10% for 1–2 mm, 15% for 2–4 mm, 21% for 4–8 mm, and 29% for 8–16 mm. Furthermore, the limestone aggregate mixture exhibited an average saturated surface dry specific gravity of 2.69 g/cm^3^, a water absorption ratio of 1.2%, and a Los Angeles abrasion loss value of 25%. Microscopic analyses were conducted on the limestone aggregate specimens for comprehensive material characterization.

To investigate the fundamental microstructure of materials, sophisticated instruments utilizing electronic and optical systems have been developed to enable high-magnification processing and analysis, thereby revealing intricate details. Scanning Electron Microscopy (SEM) operates by scanning a material’s surface with a focused beam of high-energy electrons. This technique is widely favored due to its capacity for three-dimensional imaging and elemental composition analysis. Consequently, measurements acquired via SEM exhibit enhanced reliability compared to conventional methodologies [55,56].

Scanning Electron Microscopy (SEM) coupled with Energy Dispersive X-ray (EDX) microanalysis was employed to investigate the microstructure of the limestone aggregate. SEM imaging was conducted at a magnification of ×20,000. Microstructural examination of the aggregate revealed a low porosity matrix with observed micro-cracks exhibiting limited thickness. Notably, the detection of elevated calcium (Ca) concentrations suggests the potential durability of the limestone aggregate (Figure 1b). Based on the SEM analysis, it is inferred that the limestone aggregate may possess superior durability compared to numerous other concrete aggregates, a finding supported by prior research [57,58]. Furthermore, Fourier-Transform Infrared Spectroscopy (FTIR) analysis of the aggregate demonstrated a significant spectral variation beyond 1000 cm^−1^. This fluctuation is attributed to variations in carbon–oxygen bond density. The FTIR analysis indicates that the tested limestone aggregate exhibits sufficient durability for application in concrete (Figure 1b).

In this experimental investigation, potable water conforming to the TS EN 1008 standard [59] was utilized as the mixing water. CEM I 42.5 R Portland cement supplied by Elazig in Türkiye Cement Factory (Kestel, Türkiye) was used as the binding material. To assess the influence of the water-to-cement (*W*/*C*) ratio on the compressive strength characteristics of concrete, *W*/*C* ratios of 0.20, 0.25, 0.30, 0.35, and 0.40 were evaluated.

### 2.2. Experimental Method

**Figure 2:**
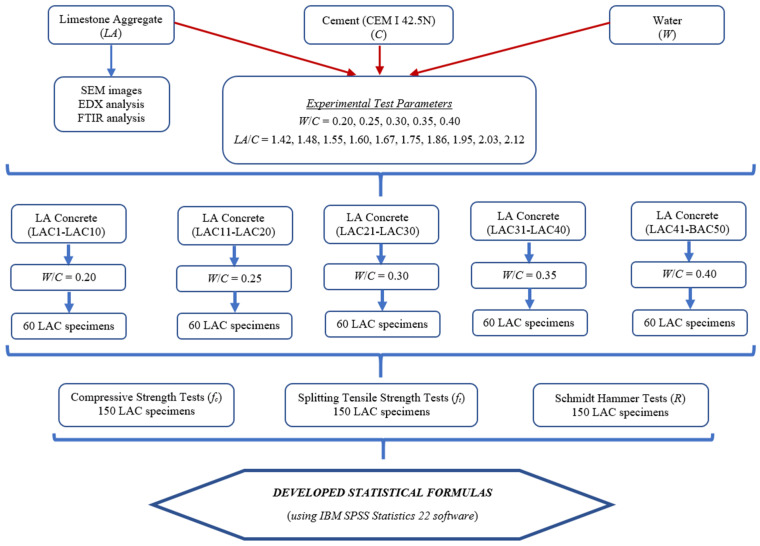
Flowchart depicting the experimental design and statistical analysis procedure.

This study investigated the influence of varying water-to-cement (*W*/*C*) and limestone aggregate-to-cement (*LA*/*C*) ratios on concrete strength. Specifically, concrete mixtures were prepared with *W*/*C* ratios of 0.20 (LAC1-LAC10), 0.25 (LAC11-LAC20), 0.30 (LAC21-LAC30), 0.35 (LAC31-LAC40), and 0.40 (LAC41-LAC50). For each *W*/*C* ratio, ten distinct *LA*/*C* combinations were evaluated, alongside three control specimens. A total of 300 concrete cubes (150 mm × 150 mm × 150 mm) were fabricated: 150 for compressive strength testing and 150 for splitting tensile strength testing. Prior to mechanical testing, the Schmidt hammer rebound hardness test was conducted at ten randomly selected locations on each specimen. The average rebound value (*R*) was recorded for each specimen. A comprehensive flowchart detailing the experimental methodology is presented in Figure 2. In this figure, *LA* represents the limestone aggregate content (kg/m^3^), *C* denotes the cement content (kg/m^3^), and *W* indicates the water content (kg/m^3^) within the concrete mixtures.

**Figure 3:**
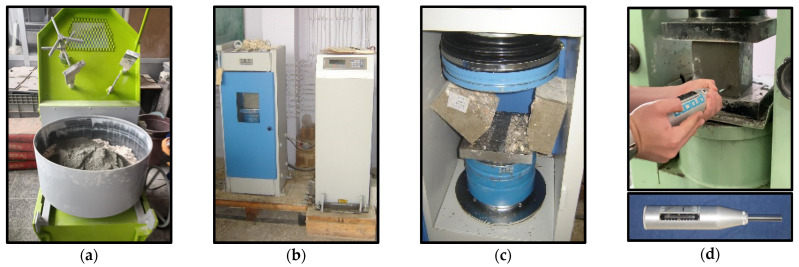
Views of experimental tests: (**a**) concrete mixer, (**b**) concrete compressive strength test, (**c**) concrete splitting tensile strength test, (**d**) Schmidt surface hardness test.

For each concrete batch, limestone aggregate, cement, and water were precisely weighed and prepared prior to mixing. A 125 L capacity, horizontal-axis laboratory mixer was employed for concrete mixing (Figure 3a).

Based on practical experience, the sequence of material introduction into the mixer and the mixing protocol significantly influence the homogeneity of the resulting concrete [60]. Fresh concrete was cast into 150 mm × 150 mm × 150 mm molds following the guidelines of ASTM C 192 [61] for subsequent testing. To ensure uniform compaction within the molds, concrete was placed in two distinct layers. After each layer was poured, a vibration was applied using a vibrating table at consistent intervals.

#### 2.2.1. Concrete Compressive Strength Test

To evaluate the influence of varying water-to-cement (*W*/*C*) and limestone aggregate-to-cement (*LA*/*C*) ratios on the compressive strength of the produced limestone aggregate concretes, 150 mm × 150 mm × 150 mm cubic specimens were subjected to compressive strength testing in accordance with TS EN 12390-3 [62]. A 2500 kN capacity concrete testing press was utilized for this purpose (Figure 3b). Specimens were loaded at a constant stress rate of 6.8 MPa/s until failure, and the ultimate loads were recorded. The compressive strengths were then calculated using Equation (1).

(1) $$f_{c}=\frac{P}{A}$$

In Equation (1), *f_c_* = compressive strength (MPa), *P* = maximum load (N) that causes the specimen to fracture, and *A* = cross-sectional area (mm^2^) of the specimen perpendicular to the direction of load application.

#### 2.2.2. Concrete Splitting Tensile Strength Test

In a manner analogous to the compressive strength test, cubic specimens of limestone aggregate concrete, maintained under identical conditions and possessing equivalent dimensions, underwent a splitting tensile strength test conforming to the TS EN 12390-6 standard [63] (Figure 3c). The specimens were subjected to a constant loading rate of 1.05 MPa/s, and the failure loads were recorded. Subsequently, the splitting tensile strength was calculated using Equation (2). The compressive and splitting tensile strength values derived from this investigation are presented as the arithmetic mean of three concrete specimens for each series within the experimental results section.

(2) $$f_{t}=\frac{2\;P}{\pi \;D\;L}\;\;$$

In Equation (2), *f_t_* = splitting tensile strength (MPa), *P* = compressive load causing fracture (N), *D* = diameter of the cubic specimen (mm), and *L* = length of the cubic specimen (mm).

#### 2.2.3. Schmidt Hammer Rebound Hardness Test

The surface hardness of 150 mm concrete cubes, cured for 28 days, was evaluated using a Schmidt hammer in accordance with TS EN 12504-2 and ASTM C 805M-13a standards [64,65]. Prior to testing, the Schmidt hammer was calibrated to ensure accuracy. To mitigate experimental variability, specimens were secured under a compression press during testing (Figure 3d). For each series, three specimens were tested, with ten measurements recorded per specimen. The reported surface hardness values represent the arithmetic mean of the resulting thirty measurements.

Previous literature has demonstrated the use of impact energy of 2.207 Nm, as specified by ISRM 2007 [66], on rock materials with compressive strengths ranging from 20 to 150 MPa. The Schmidt hammer tests, conducted perpendicular to the projection plane, adhered to ISRM 2014 [66] recommended procedures. To ensure accurate results, the test surface was prepared to be smooth and free of dust. This test serves to determine a rebound number (*R*) that represents specimen durability. The Schmidt hammer test involves a spring-loaded steel mass impacting the test surface, with the resulting rebound of the mass from the piston being measured. The rebound number (*R*) is then used as an index of specimen durability [66,67].

### 2.3. Statistical Analysis

This study utilizes statistical methodologies for the analysis of experimental data. IBM SPSS Statistics 22, a robust statistical software package, was employed for data analysis, management, and visualization. This software facilitated the application of various statistical techniques, including modeling, regression analysis, and outlier detection [68,69]. Within the statistical framework of this study, compressive strength (*f_c_*) and splitting tensile strength (*f_t_*) were defined as output variables, while water-to-cement ratio (*W*/*C*), limestone aggregate-to-cement ratio (*LA*/*C*), and rebound number (*R*) were defined as input variables. Through iterative analysis, linear equations were determined to provide the most reliable predictive models for *f_c_* and *f_t_*. The coefficients for these equations were derived using SPSS Statistics 22.

## 3. Experimental Results

Following a 28-day curing period, compressive strength (*f_c_*), splitting tensile strength (*f_t_*), and Schmidt hammer rebound number (*R*) tests were conducted on a series of limestone aggregate concrete specimens. Experimental values for *f_c_*, *f_t_*, and *R* were recorded. Statistical analyses were then performed to evaluate the influence of varying water-to-cement ratios (*W*/*C*) and limestone aggregate-to-cement ratios (*LA*/*C*) on these experimental outcomes. Table 1 presents the dimensionless parameters and corresponding experimental results for the 150 mm × 150 mm × 150 mm limestone aggregate concrete cubic specimens. To account for the inherent variability among the three specimens tested per series, Table 1 also includes the absolute relative deviation (ARD) values, calculated from the average *f_c_*, *f_t_*, and *R* measurements.

The cement content of the tested concrete specimens was maintained at a constant value of 300 kg/m^3^. The water content within 1 m^3^ of fresh concrete varied across the specimen series: 60 kg for LAC1-LAC10, 75 kg for LAC11-LAC20, 90 kg for LAC21-LAC30, 105 kg for LAC31-LAC40, and 120 kg for LAC41-LAC50. Correspondingly, the aggregate content within 1 m^3^ of fresh concrete for each series was 636, 609, 585, 558, 525, 501, 480, 465, 444, and 426 kg, respectively.

**Figure 4:**
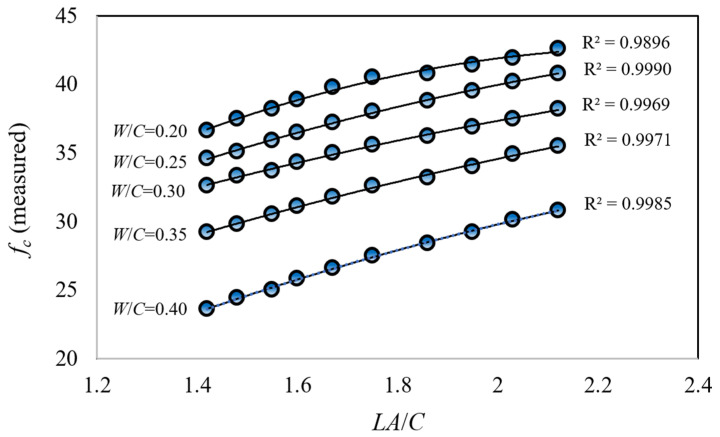
Variation of experimentally measured concrete compressive strength values (*f_c_*) with aggregate-to-cement ratio (*A*/*C*) for constant water-to-cement ratios (*W*/*C*).

Analysis of the relationship between concrete compressive strength (*f_c_*) and the limestone aggregate-to-cement ratio (*LA*/*C*) in produced limestone aggregate concrete cubic specimens revealed a near-linear correlation, evidenced by a coefficient of determination approaching unity (Figure 4). The findings demonstrated an inverse relationship between the water-to-cement ratio (*W*/*C*) and *f_c_* values, while a direct relationship was observed between the *LA*/*C* ratio and *f_c_* values. Specifically, an increase in the *W*/*C* ratio from 0.20 to 0.25 resulted in an average decrease of approximately 5.6% in *f_c_* values. Similarly, increases from 0.25 to 0.30, 0.30 to 0.35, and 0.35 to 0.40 in the *W*/*C* ratio led to average *f_c_* value reductions of approximately 6.1%, 8.7%, and 15.9%, respectively. Overall, an increase in the *W*/*C* ratio from 0.20 to 0.40 corresponded to an approximate 31.8% decrease in *f_c_* values, highlighting the significant influence of the *W*/*C* ratio on concrete strength. Conversely, an approximate 50% increase in the *LA*/*C* ratio, from 1.42 to 2.12, resulted in an average increase of approximately 18% in *f_c_* values.

**Figure 5:**
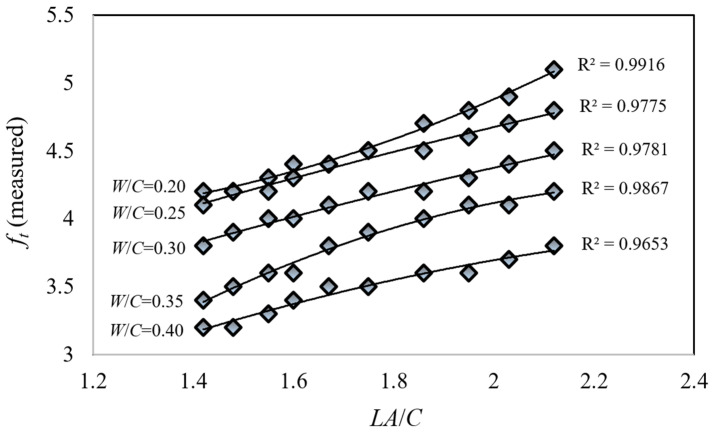
Variation of experimentally measured concrete splitting tensile strength values (*f_t_*) with aggregate-to-cement ratio (*A*/*C*) for constant water-to-cement ratios (*W*/*C*).

Upon examination of the splitting tensile strength values (*f_t_*) of limestone aggregate concrete cubic specimens produced in Figure 5, with respect to changes in the limestone aggregate-to-cement ratio, a near-linear relationship is observed, similar to the trend in concrete compressive strength. The coefficient of determination is notably close to 1. It was determined that an increase in the water-to-cement ratio resulted in a decrease in *f_t_* values, while an increase in the limestone aggregate-to-cement ratio led to an increase in *f_t_* values. Specifically, an increase in the water-to-cement ratio from *W*/*C* = 0.20 to *W*/*C* = 0.25 resulted in an average decrease of approximately 2.6% in *f_t_* values. Similarly, an increase from *W*/*C* = 0.25 to *W*/*C* = 0.30 led to an average decrease of approximately 6.6%, and an increase from *W*/*C* = 0.30 to *W*/*C* = 0.35 resulted in an average decrease of approximately 7.7%. Furthermore, an increase from *W*/*C* = 0.35 to *W*/*C* = 0.40 resulted in an average decrease of approximately 8.9%. Overall, an increase in the water-to-cement ratio from *W*/*C* = 0.20 to *W*/*C* = 0.40 resulted in an average decrease of approximately 23.5% in *f_t_* values. This clearly demonstrates the significant impact of the water-to-cement ratio on concrete strength. It was calculated that the concrete splitting tensile strength increased by approximately 19% on average as the limestone aggregate-to-cement ratio increased from 1.42 to 2.12 (approximately 50%). It was determined that both *f_c_* and *f_t_* values exhibited approximately similar rates of change with respect to the limestone aggregate-to-cement ratio.

**Figure 6:**
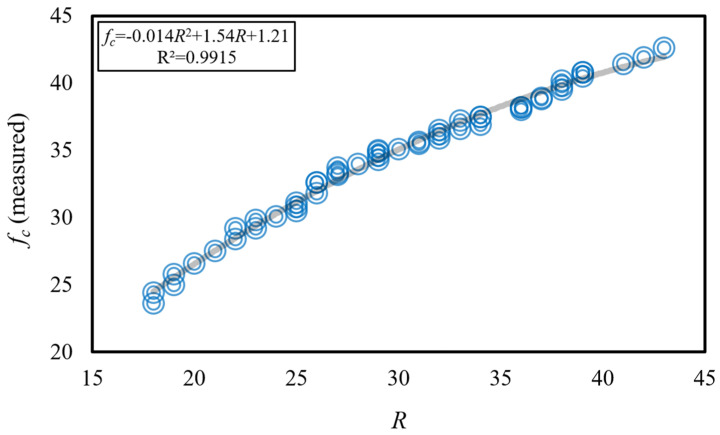
Variation of experimentally measured *f_c_* values with Schmidt hammer rebound value.

**Figure 7:**
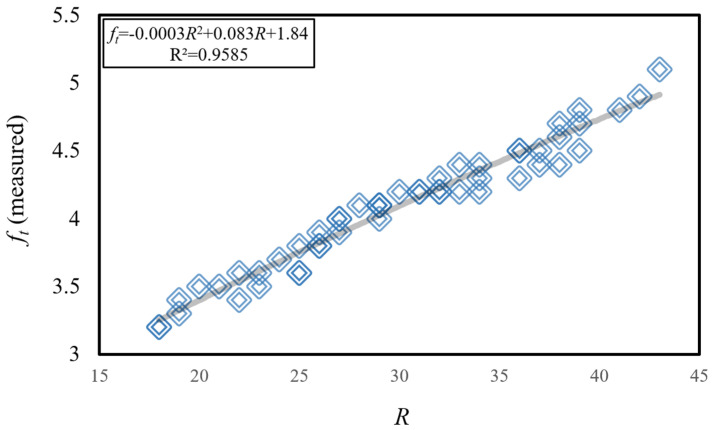
Variation of experimentally measured *f_t_* values with Schmidt hammer rebound value.

The correlation between the compressive strength values (*f_c_*) of limestone aggregate concrete specimens and the Schmidt hammer rebound value (*R*) is illustrated in Figure 6, while Figure 7 depicts the correlation between the splitting tensile strength values (*f_t_*) of the same specimens and the Schmidt hammer rebound value (*R*). Upon examination of the experimental findings in Figure 6, it was observed that the lowest *R* value (*R* = 18 N) and the lowest *f_c_* value (*f_c_* = 23.6 MPa) were measured for the highest tested water-to-cement ratio (*W*/*C* = 0.40) and the lowest tested limestone aggregate-to-cement ratio (*LA*/*C* = 1.42). Conversely, the highest *R* value (*R* = 43 N) and the highest *f_c_* value (*f_c_* = 42.6 MPa) were measured for the lowest tested water-to-cement ratio (*W*/*C* = 0.20) and the highest tested limestone aggregate-to-cement ratio (*LA*/*C* = 2.12). Corresponding to the variation in Schmidt hammer rebound values between *R* = 18 N and *R* = 43 N, a variation in limestone aggregate concrete compressive strength values between *f_c_* = 23.6 MPa and *f_c_* = 42.6 MPa was observed. In comparison to the approximately 2.4-fold total increase in *R* values, a total increase of approximately 1.8-fold was measured in *f_c_* values. A strong correlation between the *f_c_* value and the *R* value was established, with a determination coefficient of R^2^ ≈ 0.99, through the derived equation *f_c_* = −0.014*R*^2^ + 1.54*R* + 1.21.

Upon examination of the experimental findings presented in Figure 7, it was observed that the lowest splitting tensile strength value (*f_t_* = 3.2 MPa) was measured for the lowest Schmidt hammer rebound value (*R* = 18 N), the highest tested water-to-cement ratio (*W*/*C* = 0.40), and the lowest tested limestone aggregate-to-cement ratio (*LA*/*C* = 1.42). Conversely, the highest splitting tensile strength value (*f_t_* = 5.1 MPa) was measured for the highest Schmidt hammer rebound value (*R* = 43 N), the lowest tested water-to-cement ratio (*W*/*C* = 0.20), and the highest tested limestone aggregate-to-cement ratio (*LA*/*C* = 2.12). While the Schmidt hammer rebound values varied within the range of from *R* = 18 N to *R* = 43 N, the limestone aggregate concrete splitting tensile strength values varied within the range of from *f_t_* = 3.2 MPa to *f_t_* = 5.1 MPa. Compared to the approximately 2.4-fold total increase in *R* values, a total increase of approximately 1.6-fold was measured in *f_t_* values. A strong correlation between the *f_t_* value and the *R* value was established, with a determination coefficient of R^2^ ≈ 0.96, through the derived equation *f_t_* = −0.0003*R*^2^ + 0.083*R* + 1.84.

## 4. Statistical Analysis Results

In the present study, the Schmidt hammer rebound values, concrete compressive strength values (*f_c_*), and concrete splitting tensile strength values (*f_t_*) of a series of limestone aggregate concrete cubic specimens were experimentally measured. Utilizing these measured values, a nonlinear regression analysis was performed for dimensionless parameters derived from cement, water, and limestone aggregate content, the water-to-cement ratio (*W*/*C*), the limestone aggregate-to-cement ratio (*LA*/*C*), and the primary parameter of this study, the Schmidt hammer rebound value (*R*). Nonlinear equations, Equation (3) for estimating the compressive strength value of concrete (*f_c_*) and Equation (4) for estimating the splitting tensile strength value of concrete (*f_t_*), were developed. These equations were obtained through nonlinear regression analyses performed within the software program. An effort was made to determine the formula yielding a determination coefficient R^2^ as close as possible to 1.0. Following the input of dimensionless parameters, namely, *W*/*C*, *LA*/*C*, and *R*, into the program, “*f_c_*” and “*f_t_*” were defined as outputs. To ensure the calculation of “*f_c_*” and “*f_t_*” values was consistent with the experimental results, the coefficients of the relevant parameters were determined by the program. The program outputs and standard error values of the relevant dimensionless parameters obtained from the software program are presented in Table 2.

(3) $$f_{c}=33.15\times \left(\frac{W}{C}\right)-4.89\times \left(\frac{LA}{C}\right)+1.10\times R$$

(4) $$f_{t}=4.36\times \left(\frac{W}{C}\right)-0.37\times \left(\frac{LA}{C}\right)+0.11\times R$$

**Figure 8:**
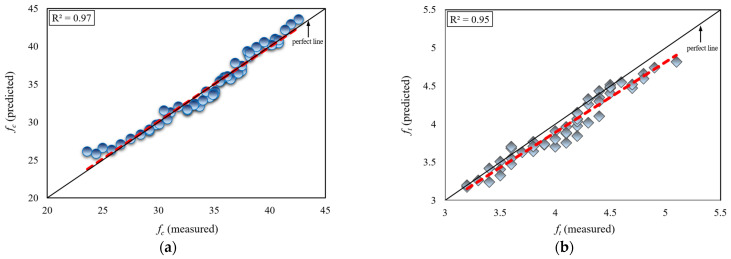
Comparison of the measured and predicted limestone aggregate concrete strength values: (**a**) for *f_c_*, (**b**) for *f_t_.*

In the present study, the accuracy of Equation (3), developed for the estimation of compressive strength values of the tested concrete cubic specimens, and Equation (4), developed for the estimation of splitting tensile strength values, was investigated through regression analysis (Figure 8). To this end, Figure 8a presents the comparison between the concrete compressive strength values “*f_c_* (predicted)”, calculated using Equation (3), and the experimentally measured concrete compressive strength values “*f_c_* (measured).” Similarly, Figure 8b presents the comparison between the concrete splitting tensile strength values “*f_t_* (predicted)”, calculated using Equation (4), and the experimentally measured concrete splitting tensile strength values “*f_t_* (measured).

The determination coefficients for Equation (3), developed for the purpose of estimating the compressive strength of concrete, and Equation (4), developed for the purpose of estimating the splitting tensile strength of concrete, were calculated as R^2^ = 0.97 and R^2^ = 0.95, respectively. Experimental findings were plotted on the x-axis, while statistical findings were plotted on the y-axis. The curve fitted to the data nearly coincides with the ideal line (i.e., the 45° line). This demonstrates that the statistical results are in strong agreement with the experimental results. Consequently, the reliable applicability of Equations (3) and (4), developed in the present study for the strength of conventional concrete produced using varying Schmidt hammer rebound values (*R*) and differing concrete content ratios (*W*/*C* and *LA*/*C*), is substantiated.

**Figure 9:**
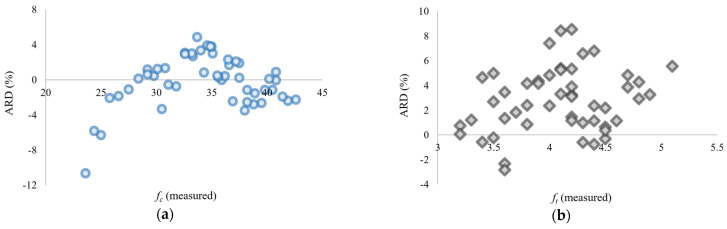
Absolute relative deviation (ARD, %) calculated for concrete strength values predicted by developed statistical methods: (**a**) for *f_c_*, (**b**) for *f_t_.*

To demonstrate the reliable applicability of Equations (3) and (4), the variation of measured strength values with absolute relative deviation (ARD) is presented (Figure 9). In this context, examination of the absolute relative deviation (ARD, %) values, calculated using Equation (5) [70,71], for the experimentally measured fc values from the present study, as shown in Figure 9a, reveals an average deviation of approximately 2.1%. Similarly, examination of the ARD values for the experimentally measured *f_t_* values, calculated using Equation (5) and presented in Figure 9b, indicates an average deviation of approximately 3.0%. Consequently, it can be asserted that the deviation magnitudes are closely aligned with the zero axis. This evaluation substantiates the strong compatibility between the experimental and statistical results, leading to the conclusion that Equations (3) and (4), developed within this study, can be employed with confidence.

(5) $$ARD\;\left(\%\right)=\frac{experimental\;data-model\;data}{experimental\;data}\times 100$$

## 5. Discussion of Statistical Methods Developed from Previous Study from the Literature and Present Study

**Figure 10:**
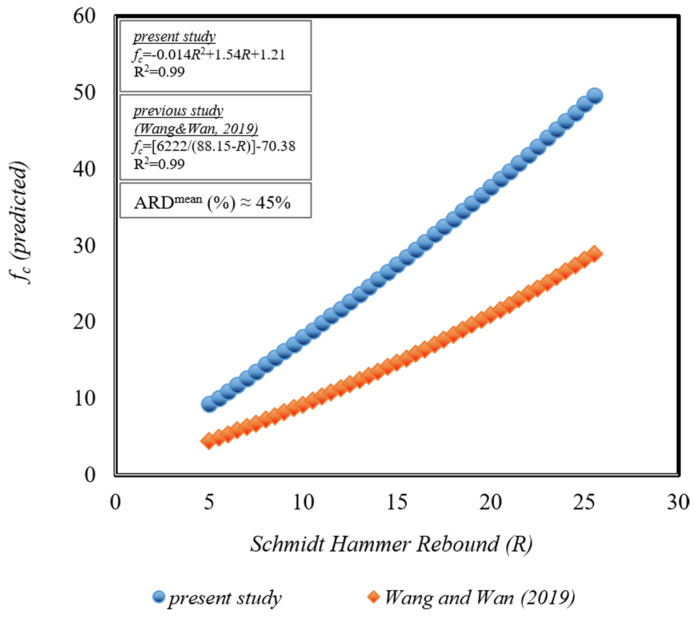
Comparison of the changes in *R* values with the *f_c_* values calculated by the statistical method from the previous study in the literature [72] and the present study.

Figure 10 illustrates the variation in concrete compressive strength values (*f_c_*) predicted using statistical methods developed with Schmidt hammer rebound (*R*) values. Given the unknown aggregate, cement, and water content and ratios in existing older structures, this section focuses solely on developing the equation relating the Schmidt hammer rebound (*R*) value to the concrete compressive strength (*f_c_*) value. To this end, only the data from Equation (6) of the present study, where the R value was used as input and the fc value as output, were compared with Equation (7) from a previous study in the literature [72] (Figure 10). The relevant equations were evaluated by calculating fc for the Schmidt hammer rebound value range of *R* = 5–25 N. Consequently, when values within the range of R = 5–25 N were considered, *f_c_* values ranging from 9.26 to 49.58 MPa were obtained using Equation (6) from the present study, and *f_c_* values ranging from 4.45 to 28.93 MPa were obtained using Equation (7) from the previous study in the literature [72].

(6) $$f_{c}=-0.014R^{2}+1.54R+1.21$$

(7) $$f_{c}=\frac{6222}{\left(88.15-R\right)}-70.38$$

Given that concrete standards typically propose distinct equations for strength ranges *f_c_* < 50 MPa or *f_c_* > 50 MPa, the comparison herein is limited to *f_c_* < 50 MPa. It is observed that the curves fitted using the statistical method from the present study and the statistical method from the previous literature study exhibit near-linear trends (Figure 10). Both equations demonstrate high determination coefficients. The absolute relative deviation value is calculated as ARD (%) ≅ 46%. Despite the close proximity of the fit, significance coefficients, and slopes of the fitted curves between both equations, the elevated absolute relative deviation ratio (ARD) can be attributed to the present study’s equation being designed to predict higher compressive strengths.

## 6. Conclusions

This study discusses and presents experimental findings pertaining to concrete compressive strength, concrete splitting tensile strength, and Schmidt hammer rebound hardness tests conducted on a series of limestone aggregate concretes. Statistical methods for estimating *f_c_* and *f_t_* were developed using the tested dimensionless parameters (*W*/*C* and *LA*/*C*) and the Schmidt hammer rebound value (*R*). The principal conclusions drawn from this research are presented below:

- An investigation revealed that increasing the water-to-cement ratio (*W*/*C*) from 0.20 to 0.40 resulted in a mean reduction of approximately 31.8% in concrete compressive strength (*f_c_*) and a mean reduction of approximately 23.5% in concrete splitting tensile strength (*f_t_*). These findings underscore the significant influence of the water-to-cement ratio on concrete strength development.
- Variations in Schmidt hammer rebound values, ranging from *R* = 18 N to 43 N, correlated with changes in the compressive strength (*f_c_*) of limestone aggregate concrete within the range of from 23.6 MPa to 42.6 MPa. Concurrently, the splitting tensile strength (*f_t_*) of the concrete specimens exhibited variations within the range of from 3.2 MPa to 5.1 MPa.
- An approximately 2.4-fold increase in Schmidt hammer rebound (*R*) values was observed, corresponding to an approximately 1.8-fold increase in compressive strength (*f_c_*) and an approximately 1.6-fold increase in splitting tensile strength (*f_t_*).
- A strong correlation was observed between compressive strength (*f_c_*) and Schmidt hammer rebound (*R*) values, as evidenced by a coefficient of determination (R^2^) of approximately 0.99. Similarly, a strong correlation was found between splitting tensile strength (*f_t_*) and *R* values, with an R^2^ of approximately 0.96.
- This study aims to develop an empirical formulation for predicting concrete strength based on Schmidt hammer rebound (*R*) values, applicable to both fresh concrete production and existing structural concrete assessment.
- The high-accuracy empirical formulas developed in this study, for predicting concrete compressive strength and splitting tensile strength based on Schmidt hammer rebound (*R*) values, offer a novel contribution and demonstrate the potential impact of this research on the existing literature.
- This study employed an experimental and statistical approach, comparing the results of destructive concrete tests with those obtained from the non-destructive Schmidt hammer rebound test. It is recommended that future research expand upon this work by incorporating multiple non-destructive testing methodologies.

## Figures and Tables

**Table 1:** Test parameters and related experimental findings of the tested concrete specimens.

Specimen Code	*W*/*C* (-)	*LA*/*C* (-)	*R*		*f_c_*		*f_t_*	
			Measured (N)	ARD (%)	Measured (N)	ARD (%)	Measured (N)	ARD (%)
LAC1	0.2	2.12	43	4.6	42.6	3.0	5.1	5.8
LAC2	0.2	2.03	42	3.6	41.9	0.6	4.9	5.2
LAC3	0.2	1.95	41	0.6	41.4	2.8	4.8	0.5
LAC4	0.2	1.86	39	2.9	40.8	2.1	4.7	0.4
LAC5	0.2	1.75	39	7.3	40.5	4.9	4.5	6.7
LAC6	0.2	1.67	38	6.8	39.8	7.8	4.4	4.0
LAC7	0.2	1.6	37	10.1	38.9	8.9	4.4	1.7
LAC8	0.2	1.55	36	8.5	38.2	8.0	4.3	5.8
LAC9	0.2	1.48	34	11.6	37.5	9.1	4.2	0.5
LAC10	0.2	1.42	33	6.3	36.6	7.6	4.2	1.2
LAC11	0.25	2.12	39	1.6	40.8	1.6	4.8	1.0
LAC12	0.25	2.03	38	3.4	40.2	0.7	4.7	4.1
LAC13	0.25	1.95	38	2.8	39.5	0.3	4.6	0.3
LAC14	0.25	1.86	37	0.4	38.8	8.6	4.5	2.4
LAC15	0.25	1.75	36	5.7	38.0	0.7	4.5	8.2
LAC16	0.25	1.67	33	6.3	37.2	1.6	4.4	6.7
LAC17	0.25	1.6	32	4.1	36.5	7.3	4.3	1.8
LAC18	0.25	1.55	32	7.3	35.9	9.1	4.2	1.2
LAC19	0.25	1.48	30	9.7	35.1	7.4	4.2	3.6
LAC20	0.25	1.42	29	6.7	34.6	0.2	4.1	0.4
LAC21	0.3	2.12	36	11.0	38.2	6.7	4.5	2.2
LAC22	0.3	2.03	34	7.5	37.5	6.9	4.4	2.6
LAC23	0.3	1.95	34	5.1	36.9	4.9	4.3	0.3
LAC24	0.3	1.86	32	10.1	36.2	1.0	4.2	1.0
LAC25	0.3	1.75	31	3.1	35.6	6.0	4.2	2.0
LAC26	0.3	1.67	29	2.6	35.0	7.7	4.1	2.8
LAC27	0.3	1.6	29	5.3	34.3	5.3	4.0	3.3
LAC28	0.3	1.55	27	2.7	33.7	5.5	4.0	5.0
LAC29	0.3	1.48	27	3.4	33.3	3.5	3.9	3.7
LAC30	0.3	1.42	26	4.5	32.6	3.1	3.8	5.2
LAC31	0.35	2.12	31	6.0	35.5	6.1	4.2	2.0
LAC32	0.35	2.03	29	1.2	34.9	5.5	4.1	0.1
LAC33	0.35	1.95	28	2.1	34.0	3.1	4.1	1.1
LAC34	0.35	1.86	27	0.4	33.2	4.1	4.0	3.3
LAC35	0.35	1.75	26	0.1	32.6	7.7	3.9	4.2
LAC36	0.35	1.67	26	0.8	31.8	4.5	3.8	7.6
LAC37	0.35	1.6	25	0.6	31.1	2.1	3.6	0.2
LAC38	0.35	1.55	25	1.6	30.5	2.9	3.6	5.4
LAC39	0.35	1.48	23	1.2	29.8	2.7	3.5	6.9
LAC40	0.35	1.42	22	0.1	29.2	0.8	3.4	2.2
LAC41	0.4	2.12	25	0.2	30.8	5.4	3.8	0.9
LAC42	0.4	2.03	24	2.3	30.1	1.0	3.7	1.1
LAC43	0.4	1.95	23	0.8	29.2	0.7	3.6	0.3
LAC44	0.4	1.86	22	2.4	28.4	4.1	3.6	0.7
LAC45	0.4	1.75	21	1.2	27.5	3.3	3.5	1.1
LAC46	0.4	1.67	20	0.5	26.6	1.6	3.5	2.3
LAC47	0.4	1.6	19	0.2	25.8	2.6	3.4	3.1
LAC48	0.4	1.55	19	0.7	25.0	4.0	3.3	3.6
LAC49	0.4	1.48	18	0.8	24.4	0.5	3.2	0.8
LAC50	0.4	1.42	18	0.2	23.6	1.5	3.2	0.6

**Table 2:** SPSS program outputs of the developed statistical methods.

**Parameter Estimates**					
**For Equation (3)**			**For Equation (4)**		
**Parameter**	**Estimate**	**Std. Error**	**Parameter**	**Estimate**	**Std. Error**
x1	33.15	2.548	x1	4.36	0.305
x2	−4.89	0.948	x2	−0.37	0.114
x3	1.10	0.033	x3	0.11	0.004
**ANOVA ^a^**					
**For Equation (3)**			**For Equation (3)**		
**Source**	**Sum of Squares**	**df**	**Source**	**Sum of Squares**	**df**
Regression	60,459.954	3	Regression	844.021	3
Residual	38.996	47	Residual	0.559	47
Uncorrected Total	60,498.950	50	Uncorrected Total	844.580	50
Corrected Total	1186.382	49	Corrected Total	10.627	49
$R^{2}=1-\frac{Residual\;Sum\;of\;Squares}{Corrected\;Sum\;of\;Squares}=0.97$			$R^{2}=1-\frac{Residual\;Sum\;of\;Squares}{Corrected\;Sum\;of\;Squares}=0.95$		

^a^ Dependent variable: perceived usefulness.

## Data Availability

The original contributions presented in this study are included in the article. Further inquiries can be directed to the corresponding author.
